# Cardiovascular magnetic resonance characterisation of pericardial and myocardial involvement in patients with tuberculous pericardial constriction with and without HIV co-infection

**DOI:** 10.1186/1532-429X-18-S1-Q29

**Published:** 2016-01-27

**Authors:** Ntobeko A Ntusi, Gregori Palkowski, Petronella Samuels, Sulaiman Moosa, Mpiko Ntsekhe, Bongani Mayosi

**Affiliations:** 1 Division of Cardiology, Department of Medicine, University of Cape Town & Groote Schuur Hospital, Cape Town, South Africa; 2 grid.7836.a0000000419371151Cape Universities Body Imaging Centre, University of Cape Town, Cape Town, South Africa; 3 Department of Radiology, 2 Military Hospital, Cape Town, South Africa

## Background

Tuberculosis pericarditis (TBP) is the most common cause of a large pericardial effusion in the developing world, accounting for 70% of effusions in a case series from South Africa; and has a high mortality related to pericardial tamponade, constrictive pericarditis, arrhythmias and heart failure. Manifestations of TBP include pericarditis with pericardial effusion, effusive-constrictive and constrictive pericarditis. There has been a dramatic resurgence in TBP in the context of co-infection with the human immunodeficiency virus (HIV). Almost 100% of pericardial effusions in those infected with HIV in sub-Saharan Africa are due to tuberculosis, compared with 50-70% in those HIV-uninfected and less than 5% in industrial nations. In patients with TBP, co-infection with HIV is associated with increased heart failure, haemodynamic instability, electrocardiographic (ECG) ST elevation and mortality, suggesting an aggressive myopericarditis in the context of HIV co-infection. However, little is known about myocardial involvement in patients with TBP. Cardiovascular magnetic resonance (CMR) can assess non-invasively cardiac function, myocardial oedema, inflammation and fibrosis. We hypothesised that HIV co-infection would be associated with increased myocardial pathology on CMR in patients with TBP.

## Methods

The purpose of this study was to assess cardiac and pericardial structure and function in patients with TBP with and without HIV co-infection and to assess the relationship of LV function with other imaging biomarkers. 72 patients with TBP (37 male (51.3%), mean age 40 ± 14.3) were included in the study. Of these, 35 were HIV-infected (17 male (48.6%), mean age 34 ± 8) and 37 were HIV-uninfected (20 male (54.1%), mean age 51 ± 16). Assessments included clinical examination, ECG, echocardiography, serum and pericardial biomarkers and CMR (biventricular volumes and function, oedema, and late gadolinium enhancement - LGE).

## Results

HIV-infected TBP patients were younger (p < 0.001), had lower serum haemoglobin (p < 0.001) and were more likely to have NYHA class III and IV symptoms (p < 0.001). There were no differences on ECG and echocardiography between HIV-infected and -uninfected TBP patients. There were also no differences in global systolic function and myocardial signal intensity ratio on STIR imaging between HIV-infected and -uninfected TBP patients. Focal fibrosis on LGE was found more commonly in those with HIV infection (p < 0.001). Pericardial effusions were frequent (>50%) in both groups of TBP patients. Determinants of LV ejection fraction in TBP included heart rate, LV size, E/A ratio, pericardial LGE and pericardial thickness (all p < 0,01).

## Conclusions

HIV co-infection is associated with increased focal myocardial fibrosis in TBP patients suggesting increased myocardial inflammation in those with HIV co-infection. In the future, it will be important to assess the prognostic significance of these data.

